# Disability, psychological distress and quality of life in relation to cancer diagnosis and cancer type: population-based Australian study of 22,505 cancer survivors and 244,000 people without cancer

**DOI:** 10.1186/s12916-020-01830-4

**Published:** 2020-12-01

**Authors:** Grace Joshy, Joanne Thandrayen, Bogda Koczwara, Phyllis Butow, Rebekah Laidsaar-Powell, Nicole Rankin, Karen Canfell, John Stubbs, Paul Grogan, Louise Bailey, Amelia Yazidjoglou, Emily Banks

**Affiliations:** 1 National Centre for Epidemiology and Population Health, Research School of Population Health, Australian National University, Mills Road, Acton, Canberra, ACT 2601 Australia; 2 Flinders University and Flinders Medical Centre, Adelaide, SA Australia; 3 Centre for Medical Psychology and Evidence-based Medicine, School of Psychology, Faculty of Science, The University of Sydney, Sydney, NSW Australia; 4 Cancer Research Division, Cancer Council New South Wales, Kings Cross, NSW Australia; 5 Prince of Wales Clinical School, University of New South Wales, Sydney, NSW Australia; 6 CanSpeak Australia, Sydney, NSW Australia; 7 Primary Care Collaborative Cancer Clinical Trials Group Community Advisory Group, Melbourne, VIC Australia; 8 Psycho-oncology Cooperative Research Group Community Advisory Group, Camperdown, NSW Australia; 9 Sax Institute, Haymarket, NSW Australia

**Keywords:** Survivorship, Cancer survivor, Disability, Psychological distress, Quality of life, Australian, Cohort

## Abstract

**Background:**

Improved survival means that cancer is increasingly becoming a chronic disease. Understanding and improving functional outcomes are critical to optimising survivorship. We quantified physical and mental health-related outcomes in people with versus without cancer, according to cancer type.

**Methods:**

Questionnaire data from an Australian population-based cohort study (45 and Up Study (*n* = 267,153)) were linked to cancer registration data to ascertain cancer diagnoses up to enrolment. Modified Poisson regression estimated age- and sex-adjusted prevalence ratios (PRs) for adverse person-centred outcomes—severe physical functional limitations (disability), moderate/high psychological distress and fair/poor quality of life (QoL)—in participants with versus without cancer, for 13 cancer types.

**Results:**

Compared to participants without cancer (*n* = 244,000), cancer survivors (*n* = 22,505) had greater disability (20.6% versus 12.6%, respectively, PR = 1.28, 95%CI = (1.25–1.32)), psychological (22.2% versus 23.5%, 1.05 (1.02–1.08)) and poor/fair QoL (15.2% versus 10.2%; 1.28 (1.24–1.32)). The outcomes varied by cancer type, being worse for multiple myeloma (PRs versus participants without cancer for disability 3.10, 2.56–3.77; distress 1.53, 1.20–1.96; poor/fair QoL 2.40, 1.87–3.07), lung cancer (disability 2.81, 2.50–3.15; distress 1.67, 1.46–1.92; poor/fair QoL 2.53, 2.21–2.91) and non-Hodgkin’s lymphoma (disability 1.56, 1.37–1.78; distress 1.20, 1.05–1.36; poor/fair QoL 1.66, 1.44–1.92) and closer to those in people without cancer for breast cancer (disability 1.23, 1.16–1.32; distress 0.95, 0.90–1.01; poor/fair QoL 1.15, 1.05–1.25), prostate cancer (disability 1.11, 1.04–1.19; distress 1.09, 1.02–1.15; poor/fair QoL 1.15, 1.08–1.23) and melanoma (disability 1.02, 0.94–1.10; distress 0.96, 0.89–1.03; poor/fair QoL 0.92, 0.83–1.01). Outcomes were worse with recent diagnosis and treatment and advanced stage. Physical disability in cancer survivors was greater in all population subgroups examined and was a major contributor to adverse distress and QoL outcomes.

**Conclusions:**

Physical disability, distress and reduced QoL are common after cancer and vary according to cancer type suggesting priority areas for research, and care and support.

## Background

With improved prevention, early detection and treatment, cancer survival has increased, and cancer is increasingly becoming a chronic disease. Hence, survivors are living with cancer and/or the adverse consequences of its treatment for extended periods of time, underscoring the importance of longer-term health care outcomes of survivors including attributes central to the ability of individuals and communities to lead rich and fulfilling lives [1, 2].

These “person-centred” outcomes—including mental health, disability, social and economic participation, and quality of life—have been identified as important by cancer survivors [3, 4]. However, despite the need for evidence on these outcomes, surprisingly, little is known about them [5, 6]. Cancer is a highly heterogeneous condition, and recognition of the diversity of survivorship experiences is important. Many survivorship studies to date have involved small samples, single cancer types and short- to medium-term outcomes and/or lacked comparable individuals without cancer. Key previous large-scale studies have been restricted to older cancer survivors aged ≥ 65 years [7, 8] or used self-reported cancer only [9–12]. None has permitted large-scale integrated consideration of the full range of more common cancer types and multiple key person-centred outcomes, relative to otherwise comparable individuals without cancer. There is also a lack of reliable evidence on the joint contributions of the diagnosis of cancer and physical disability to psychological distress and quality of life, although studies have shown relationships between these factors individually [13].

This study aimed to quantify short- and long-term physical and mental health-related person-centred outcomes in people with cancer, compared to people without cancer, for a range of cancer types—overall and according to time since diagnosis, stage and recent treatment for cancer, accounting for age and sex, in a population-based Australian study of over 260,000 participants. We hypothesised that physical disability would be a major contributing factor to high psychological distress and reduced quality of life in both cancer survivors and those without cancer [13].

## Methods

The Sax Institute’s 45 and Up Study is a population-based cohort study of 267,153 men and women aged 45 and over, randomly sampled from the general population of New South Wales (NSW), Australia, using the Department of Human Services enrolment database. Participants aged 45 or over were enrolled to enable research into major diseases and health problems experienced in later life and provide reliable evidence to inform policy to support healthy ageing. The cohort includes approximately 10% of NSW residents in the eligible age group. Individuals joined the study by completing a self-administered postal questionnaire (distributed from 1 January 2006 to 31 December 2008) and giving informed consent for long-term follow-up and linkage of their data to other population health databases. The general study methods are described in detail elsewhere [14]. This study is part of an NHMRC-funded project; consumers have been involved with this project since its conception, with roles agreed between the researchers and consumers at each phase of the project (Additional file 1).

Baseline questionnaire data included self-reported information on demographic factors, medical and surgical history, height, weight, smoking, alcohol intake, physical activity, functional capacity, mental health and self-rated health and quality of life (measures described below). The study questionnaire is available at https://www.saxinstitute.org.au/our-work/45-up-study/questionnaires/.

Questionnaire data from study participants were linked probabilistically to administrative datasets including data from the NSW Central Cancer Registry (CCR, 1 January 1994 to 31 December 2013). This probabilistic matching was conducted by the NSW Centre for Health Record Linkage (CHeReL) and is known to be highly accurate (false-positive and false-negative rates < 0.4%) [15]. The linked CCR data comprised records of all diagnosed cancers (except those C44 codes that indicate a basal cell carcinoma or a squamous cell carcinoma which are not notifiable diseases thus not reported to cancer registries) for NSW residents, including the date of diagnosis and International Classification of Diseases (ICD)-coded cancer types and sites. Following the exclusion of participants with invalid data on age or date of recruitment (*n* = 461, 0.17%) or data linkage errors (*n* = 187, 0.07%), the analysis dataset consisted of 266,505 individuals.

### Exposure

The main exposure was a cancer diagnosis prior to the completion of the baseline questionnaire. Participants were classified as being a cancer survivor if they had a cancer diagnosis record in the CCR database in the 12 years prior to baseline; the type, date of diagnosis and stage of cancer were also ascertained from the CCR database. A 12-year window, based on the availability of linked data, was used to ensure a uniform probability of identification of cancer previous diagnoses from CCR database for all participants. The 12 cancer types with the highest age-standardised incidence in Australia [16] were investigated separately a priori, except cancer of the pancreas which was excluded due to the small number of cases in the 45 and Up Study; oesophagal cancer and multiple myeloma were also included due to their known adverse impact on well-being. Cancer types were classified as breast (ICD-10 AM diagnosis code C50, women only), prostate (C61, men only), lung (C33–C34), melanoma (C43), colorectal (C18–C20), non-Hodgkin’s lymphoma (NHL, C82–C86), kidney (C64), oesophagus (C15), uterus (C54–C55, women only), bladder (C67);,thyroid (C73), leukaemia (C91–C95) and multiple myeloma (C90.0) (Additional file 2: Table S1). All the remaining cancers were included in an “other cancers” category.

The time since diagnosis was classified as less than 1 year, 1 to < 5 years, 5 to < 10 years and 10 or more years. If multiple cancers were present, the diagnosis closest to the study enrolment date was used. The stage of cancer at diagnosis was classified as localised to the tissue of origin, regional spread to adjacent organs and/or regional lymph nodes, distant metastases and unknown stage (only solid cancers (ICD-10 AM diagnosis codes C00.0–C43.9 or C45.0–C80) were staged). Recent treatment was classified as yes/no based on the response to the baseline survey question, “In the last month, have you been treated for cancer?” The reference group for the study comprised respondents with no record of a cancer diagnosis in the CCR database.

### Outcomes

Physical functioning limitations were assessed using the Medical Outcomes Study Physical Functioning (MOS-PF) score [17] eliciting self-reported data on limitations in the ability to perform vigorous and moderate physical activities and tasks such as lifting or carrying shopping; climbing stairs; walking; bending, kneeling or stooping; and bathing or dressing. The MOS-PF is a valid and reliable measure of physical functioning [18], with a lower score indicating more severe functional limitation [19]. Scores ranged from 0 to 100, where higher scores represented fewer limitations, and were grouped into four categories: no limitation (MOS-PF score = 100), minor limitations (90–99), moderate limitations (60–89) and severe limitations (< 60) [14, 19].

Psychological distress was assessed using the Kessler-10 (K10), a validated measure of non-specific symptoms of psychological distress [20]. Respondents indicated the frequency of symptoms experienced in the past 4 weeks, from 1 “none of the time” to 5 “all of the time”. Scores range from 10 (no distress) to 50 (severe distress) and were categorised as low distress (10 to < 16), moderate distress (16 to < 22) and high distress (22 to 50) [21].

Self-rated health and quality of life were based on the question, “In general, how would you rate your overall health/quality of life?”, followed by response options of excellent, very good, good, fair and poor.

### Other variables

Sociodemographic and health characteristics included age (categorised as 45–64 years; 65–79 years; ≥ 80 years), gender, education (no school certificate, certificate/diploma/trade, university degree), country of birth (Australian born, not Australian born), body mass index (BMI (kg/m^2^) 15 to < 18.5, 18.5 to < 25, 25 to < 30, and ≥ 30–50), physical activity (tertiles of sessions per week weighted for intensity), smoking status (never/past/current smoker) and number of alcoholic drinks per week (0, 1–14, ≥ 15 drinks per week). The region of residence derived from the address was categorised as major city, inner regional, outer regional and remote/very remote. Comorbidities were based on responses to questions on “has a doctor ever told you that you have...”.

### Statistical methods

After logical imputation and backfilling for K10 and MOS-PF scores, we excluded those with missing data on each outcome variable (physical functioning limitations (*n* = 35,450; 13.3%), psychological distress (*n* = 30,290; 11.4%), self-rated health (*n* = 9413; 3.5%) and quality of life (*n* = 14,064; 5.3%)) from the corresponding analyses.

Descriptive statistics summarised demographic and clinical data. Modified Poisson regression models estimated prevalence ratios (PRs) and 95% confidence intervals (CIs) to quantify associations between a cancer diagnosis and each adverse person-centred outcome, categorised as binary variables: severe physical functioning limitations (MOS-PF score < 60), high/moderate psychological distress (K10 score 16–50), poor/fair self-rated health, poor/fair quality of life, overall and according to cancer type. Models were adjusted for age and sex (where applicable). Further statistical adjustments were not done as the objective was to compare prevalences and lived experiences rather than establish causality. Adjusted PRs were also estimated stratifying by clinical characteristics (time since diagnosis, stage and recent treatment). To quantify the contribution of physical disability to high psychological distress, poor/fair quality of life and poor/fair self-rated health, adjusted PRs were estimated among participants with and without a cancer diagnosis further stratified by different levels of physical functional limitations; those with neither cancer nor physical functional limitations were used as the reference group. The prevalence of severe physical functioning limitations across population subgroups was compared, separately for those with and without cancer; differences by cancer diagnosis were assessed using interaction tests.

Sensitivity analyses examined further adjustment for educational attainment, as a proxy for socioeconomic status, as well as alternative binary classification of physical functioning limitations (MOS-PF score < 90 indicating moderate/severe physical functioning limitations) and high psychological distress (K10 score 22–50 indicating high distress).

## Results

There were 22,505 cancer survivors and 244,000 people without cancer included in the analysis. Compared to participants without cancer, cancer survivors were older and more likely to be male and former smokers and were similar with respect to other characteristics examined, including levels of education, urban/rural residence, body mass index, level of physical activity and alcohol intake (Table 1).

**Table 1:** Characteristics of the study population

	Cancer survivors (*n* = 22,505)	Participants without cancer (*n* = 244,000)	Total (*n* = 266,505)
**Age group**			
45–64 years	37% (8333)	64% (155,223)	163,556
65–79 years	44% (9860)	27% (65,989)	75,849
≥ 80 years	19% (4312)	9% (22,788)	27,100
**Male**	56% (12,666)	45% (110,951)	123,617
**University degree**	19% (4302)	23% (57,161)	61,463
**Residing in major cities**	53% (11,970)	52% (127,085)	139,055
**Australian born**	78% (17,443)	75% (182,214)	199,657
**Body mass index, kg/m**^**2**^			
Overweight (25 to < 30)	38% (8482)	36% (88,825)	97,307
Obese (30 to 50)	21% (4759)	22% (52,694)	57,453
**Highest physical activity tertile**	30% (6691)	34% (82,273)	88,964
**Current smoker**	5% (1048)	7% (18,244)	19,292
**Past smoker**	41% (9308)	36% (86,885)	96,193
**≥ 15 alcoholic drinks per week**	14% (3228)	14% (34,197)	37,425
**Cardiovascular disease**	25% (5567)	17% (40,372)	45,939
**Diabetes**	11% (2583)	9% (21,307)	23,890
**Parkinson’s disease**	1% (243)	1% (1430)	1673
**Asthma**	9% (2111)	10% (25,030)	27,141
**Physical functioning limitations (MOS-PF score)**			
Median score	85	95	95
No limitation (100)	21% (4078)	35% (74,661)	78,739
Minor limitations (90–99)	27% (5151)	29% (61,215)	66,366
Moderate limitations (60–89)	31% (5885)	23% (49,437)	55,322
Severe limitations (< 60)	21% (3911)	13% (26,717)	30,628
**Psychological distress (K10 score)**			
Median score	12	12	12
Low distress (10–15)	78% (14,720)	76% (166,152)	180,872
Moderate distress (16–21)	16% (2968)	16% (34,395)	37,363
High distress (22–50)	7% (1245)	8% (16,735)	17,980
**Self-rated health**			
Excellent	8% (1808)	16% (37,057)	38,865
Very good	31% (6683)	37% (88,204)	94,887
Good	39% (8333)	33% (78,502)	86,835
Fair	18% (3917)	11%(26,950)	30,867
Poor	4% (821)	2%(4817)	5638
**Self-rated quality of life**			
Excellent	17% (3567)	24% (56,330)	59,897
Very good	34% (7277)	38% (86,841)	94,118
Good	34% (7120)	28% (64,428)	71,548
Fair	13% (2744)	9% (19,834)	22,578
Poor	2% (470)	2% (3830)	4300

Percentages are out of column totals which include missing values: education (1.7%), region of residence (1.9%), country of birth (0.8%), BMI (6.6%), physical activity (3.5%), smoking status (0.3%) and alcohol intake (2.1%). Those with missing values for an outcome are excluded from the corresponding analyses: physical functioning limitations (13.3%), psychological distress (K10 score, 11.4%), self-rated health (3.5%) and self-rated quality of life (5.3%). There were no missing values in age or sex

Of the 22,505 cancers identified, the most common were prostate (26%), breast (19%), melanoma (15%) and colorectal (13%) cancer, which accounted for nearly three quarters of all cancers (Table 2). Clinical characteristics such as time since diagnosis of cancer, cancer stage and recent treatment varied according to cancer type. The median time since diagnosis of cancer was 3.9 years, with 60% diagnosed in the 5 years prior to baseline (Table 2). Lung and oesophageal cancer survivors were more likely to have been diagnosed within the previous year compared to those with other cancers. For cancer types other than colorectal cancer, most had localised disease. The majority of survivors had not received cancer treatment in the past month, except for those with multiple myeloma (Table 2).

**Table 2:** Clinical characteristics of cancer by cancer type

	Prostate	Breast	Melanoma	Colorectal	NHL	Lung	Kidney	Uterus	Bladder	Leukaemia	Thyroid	Multiple myeloma	Oesophagus	Other cancer	Any cancer
***n***	5854	4330	3398	3011	814	493	486	479	398	376	338	183	83	2262	22,505
**Median time since diagnosis (years)**	3.4	4.9	4.5	3.8	3.6	2.1	4.0	4.0	4.4	3.7	4.0	2.9	2.2	3.7	3.9
**Time since diagnosis (years)**															
< 1	16.2%	10.3%	12.7%	16.6%	15.6%	29.8%	14.8%	15.5%	14.1%	16.8%	16.6%	17.5%	38.6%	16.8%	15.0%
1 to < 5	50.1%	41.0%	42.6%	43.7%	45.1%	45.6%	44.0%	41.5%	41.5%	48.4%	42.6%	59.6%	36.1%	44.7%	45.0%
5 to < 10	27.2%	37.6%	34.6%	31.5%	31.7%	22.1%	32.5%	34.0%	36.7%	29.0%	32.3%	20.8%	24.1%	29.5%	31.7%
10 or more	6.5%	11.1%	10.1%	8.2%	7.6%	2.4%	8.6%	9.0%	7.8%	5.9%	8.6%	2.2%	1.2%	8.9%	8.4%
**Stage**															
localised to tissue or origin	56.2%	59.6%	88.9%	41.1%	N/A	43.6%	74.9%	64.7%	54.0%	N/A	67.5%	N/A	49.4%	38.5%	55.6%
regional spread, adjacent organs and/or regional lymph nodes	6.7%	32.4%	4.5%	45.3%	N/A	25.4%	14.6%	21.3%	10.3%	N/A	15.4%		27.7%	22.7%	18.9%
distant metastases	0.7%	2.3%	1.3%	5.1%	N/A	13.4%	2.3%	3.8%	2.0%	N/A	3.0%	N/A	4.8%	10.2%	3.1%
unknown	36.5%	5.7%	5.2%	8.6%	N/A	17.7%	8.2%	10.2%	33.7%	N/A	14.2%	N/A	18.1%	28.6%	22.5%
**Recent treatment**															
No	78.7%	71.5%	92.9%	80.1%	73.8%	64.9%	87.0%	87.3%	81.2%	70.7%	85.2%	35.0%	65.1%	78.4%	79.1%
Yes	21.3%	28.5%	7.1%	19.9%	26.2%	35.1%	13.0%	12.7%	18.8%	29.3%	14.8%	65.0%	34.9%	21.6%	20.9%

Stage for any cancer includes solid cancers only (prostate, breast, melanoma, colorectal, lung, kidney, uterus, bladder, thyroid and oesophagus)

Proportions of unknown stage were 85.6%, 93.9% and 89.1% for non-Hodgkin’s lymphoma (NHL), leukaemia and multiple myeloma, respectively

Diagnosis codes grouped under “other cancers”, and the corresponding numbers of participants are included in Additional file 2: Table S1

The numbers of participants with sex-specific cancers included 28 males with breast cancer and 1 female with prostate cancer. Those were excluded from subsequent prevalence ratio analyses

**Fig. 1:**
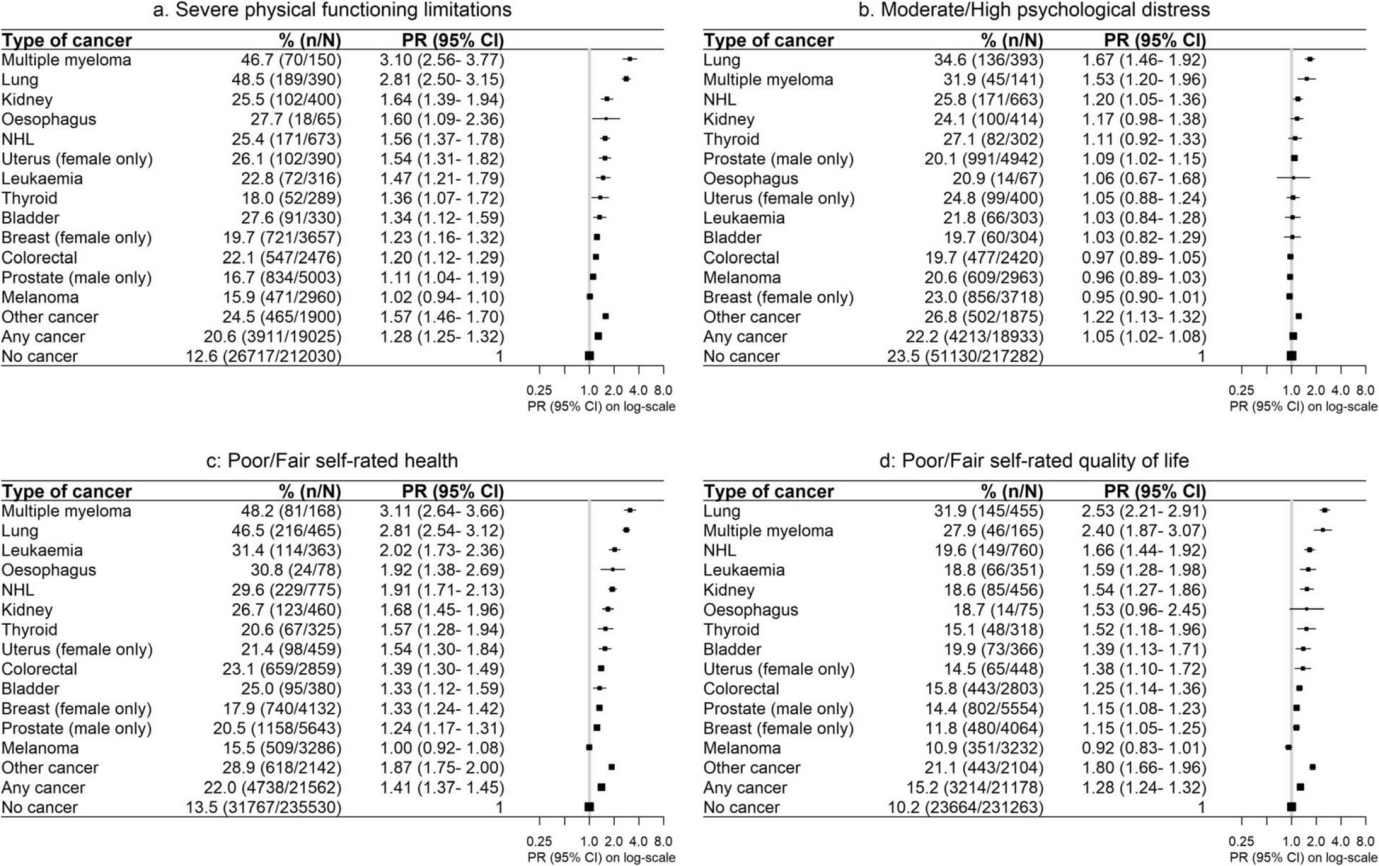
Prevalence of and age- and sex-adjusted prevalence ratios (PR) for adverse person-centred outcomes by cancer type. Numbers of cancer types may not add up to the total number for “any cancer” due to sex-specific restrictions applied to some cancer types

Overall, 21% of cancer survivors had severe physical functioning limitations, compared to 13% of people without cancer (age- and sex-adjusted PR 1.28, 95%CI 1.25–1.32) (Table 1, Fig. 1). Similarly, elevated prevalences of fair/poor self-rated health and quality of life were apparent, with PRs for cancer survivors versus those without cancer of 1.41 (1.37–1.45) and 1.28 (1.24–1.32), respectively (Fig. 1). The age- and sex-adjusted PR for moderate/high psychological distress was slightly elevated in cancer survivors (1.05, 1.02–1.08), although the crude prevalence was slightly lower (22.2% in cancer survivors versus 23.5% in people without cancer). In general, adverse person-centred outcomes were elevated for all cancer types, with the exception of some cancers in relation to psychological distress (Fig. 1), and for melanoma across all outcomes. Outcomes varied substantively by cancer type. Almost half of those with multiple myeloma and lung cancer had severe physical functioning limitations; adjusted prevalences were around threefold those of cancer-free participants (Fig. 1). They also experienced the highest levels of distress and reductions in self-rated health and quality of life. In general, participants with non-Hodgkin’s lymphoma (NHL), leukaemia and cancers of the oesophagus, uterus, bladder, thyroid and kidney had a prevalence of adverse person-centred outcomes that were below those observed in those with multiple myeloma and lung cancer and were higher than those observed for breast, colorectal and prostate cancers (Fig. 1). The composite group of less common “other cancers” (Additional file 2: Table S1) experienced consistent elevations in adverse person-centred outcomes, compared to people without cancer; PRs were at least 22% higher for all outcomes (Fig. 1).

**Fig. 2:**
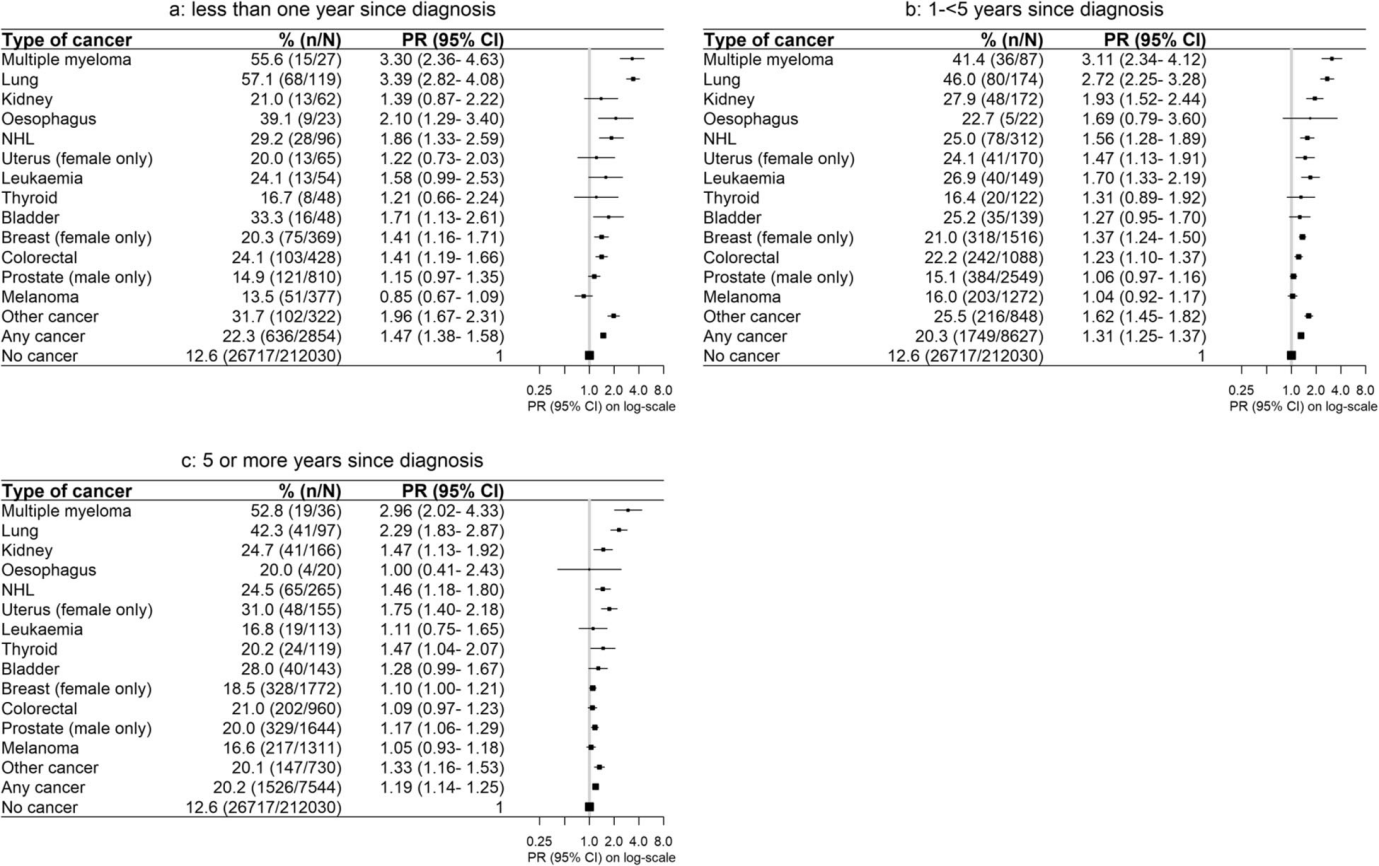
Prevalence of and age- and sex-adjusted prevalence ratios (PR) for severe physical functioning limitations by cancer type and time since diagnosis

**Fig. 3:**
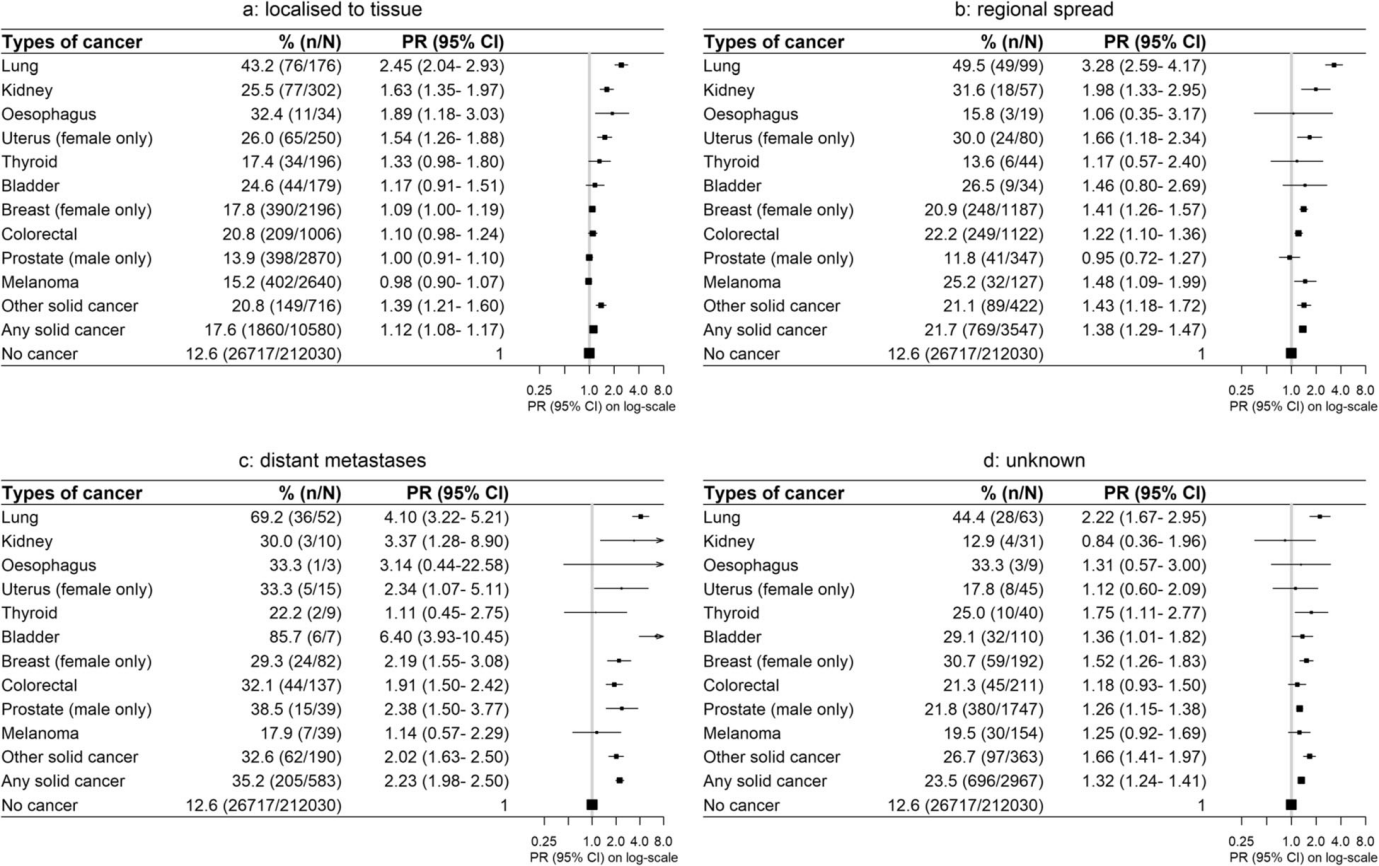
Prevalence of and age- and sex-adjusted prevalence ratios (PR) for severe physical functioning limitations by cancer type and stage

**Fig. 4:**
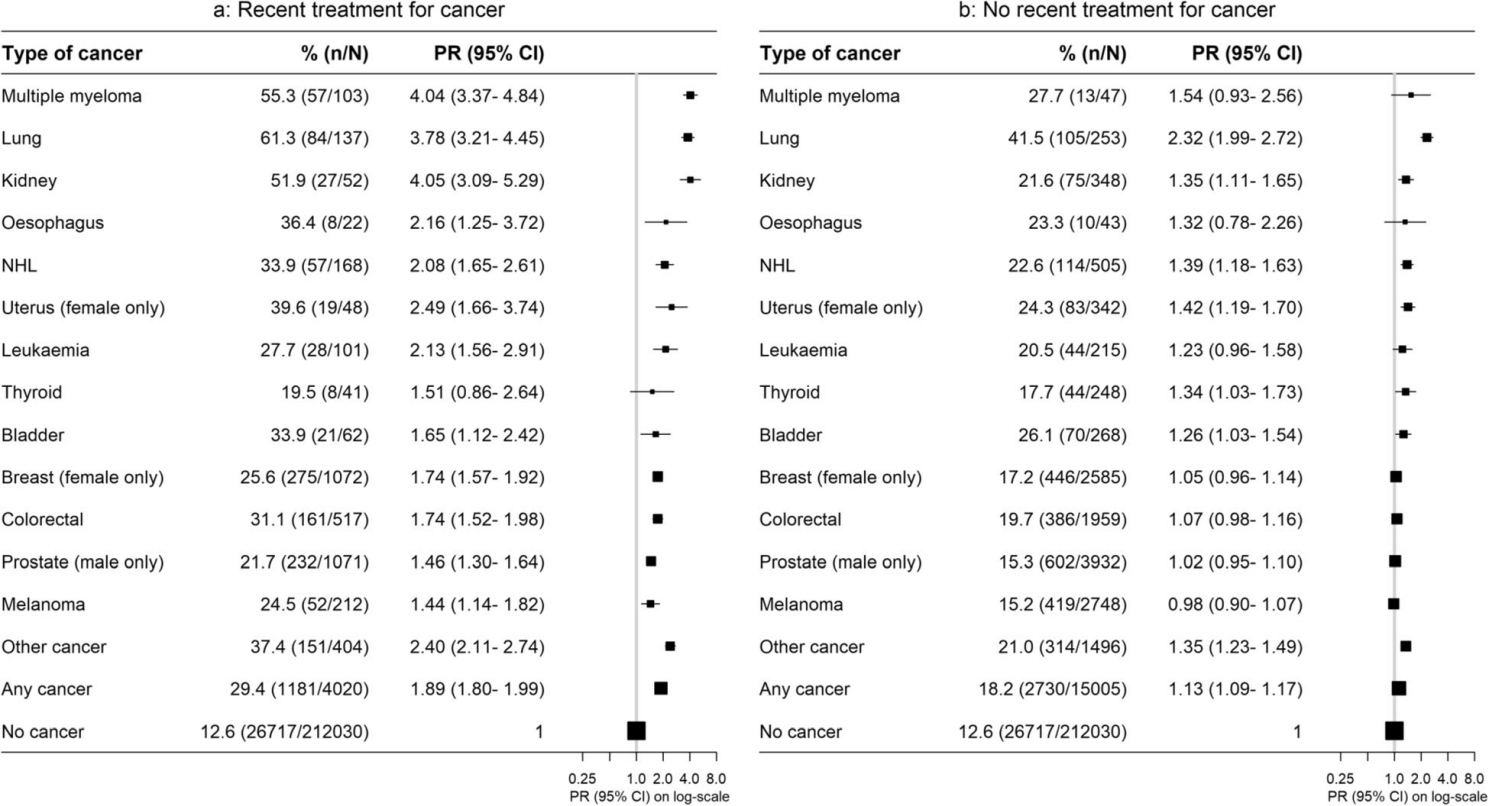
Prevalence of and age- and sex-adjusted prevalence ratios (PR) for severe physical functioning limitations by cancer type and recent treatment

Although physical functioning, self-rated health and quality of life were reduced overall for cancer survivors compared to their cancer-free peers, worse outcomes were observed with increasing recency of diagnosis, more advanced stage and treatment within the last month. Compared to cancer-free participants, the PRs for severe physical functioning limitations were 1.47 (1.38–1.58) for cancer survivors diagnosed within the previous year and 1.19 (1.14–1.25) after 5 or more years (Fig. 2). Corresponding PRs were 2.23 (1.98–2.50) for metastatic disease, 1.12 (1.08–1.17) for localised disease, 1.89 (1.80–1.99) for cancer treatment in the previous month and 1.13 (1.09–1.17) for cancer survivors not receiving treatment in the previous month (Figs. 3 and 4). Similar patterns were observed for psychological distress, self-rated health and quality of life (Additional file 2: Fig. S1-S9). However, the magnitude of the differences in psychological distress between cancer survivors and cancer-free individuals was less pronounced, and long-term survivors showed no significant elevation in psychological distress 5 or more years post-diagnosis (1.01, 0.97–1.05; Additional file 2: Fig. S1).

Within each clinical characteristic group (e.g. time since diagnosis < 1 year), there were large variations in person-centred outcomes according to cancer type, similar to those observed above; multiple myeloma and lung cancer patients experienced the worst outcomes, and patients with breast, colorectal and prostate cancer and melanoma patients had the best outcomes.

**Fig. 5:**
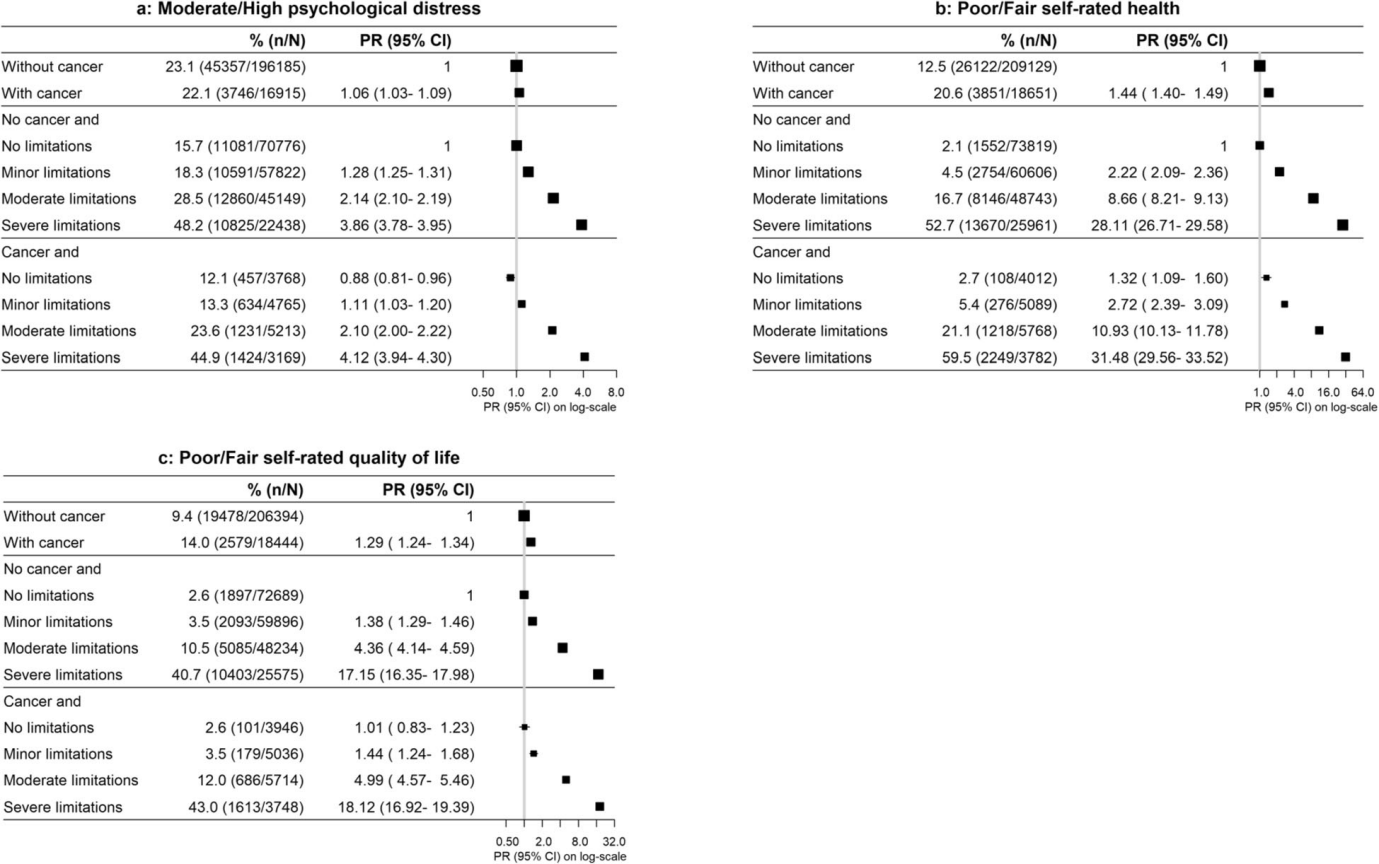
Prevalence of and age- and sex-adjusted prevalence ratios (PRs) for adverse person-centred outcomes according to joint categories of physical functioning limitations and cancer

Among individuals with and without cancer, the prevalence of moderate/high psychological distress and fair/poor self-rated health and quality of life increased markedly with increasing limitations to physical functioning (Fig. 5).

Among participants without limitations to physical functioning, compared to cancer-free individuals, participants with cancer had a significantly reduced prevalence of moderate to high psychological distress (0.88, 0.81–0.96), a significantly elevated prevalence of fair/poor self-rated health (1.32, 1.09–1.60) and no significant difference in the quality of life (1.01, 0.83–1.23; Fig. 5).

**Fig. 6:**
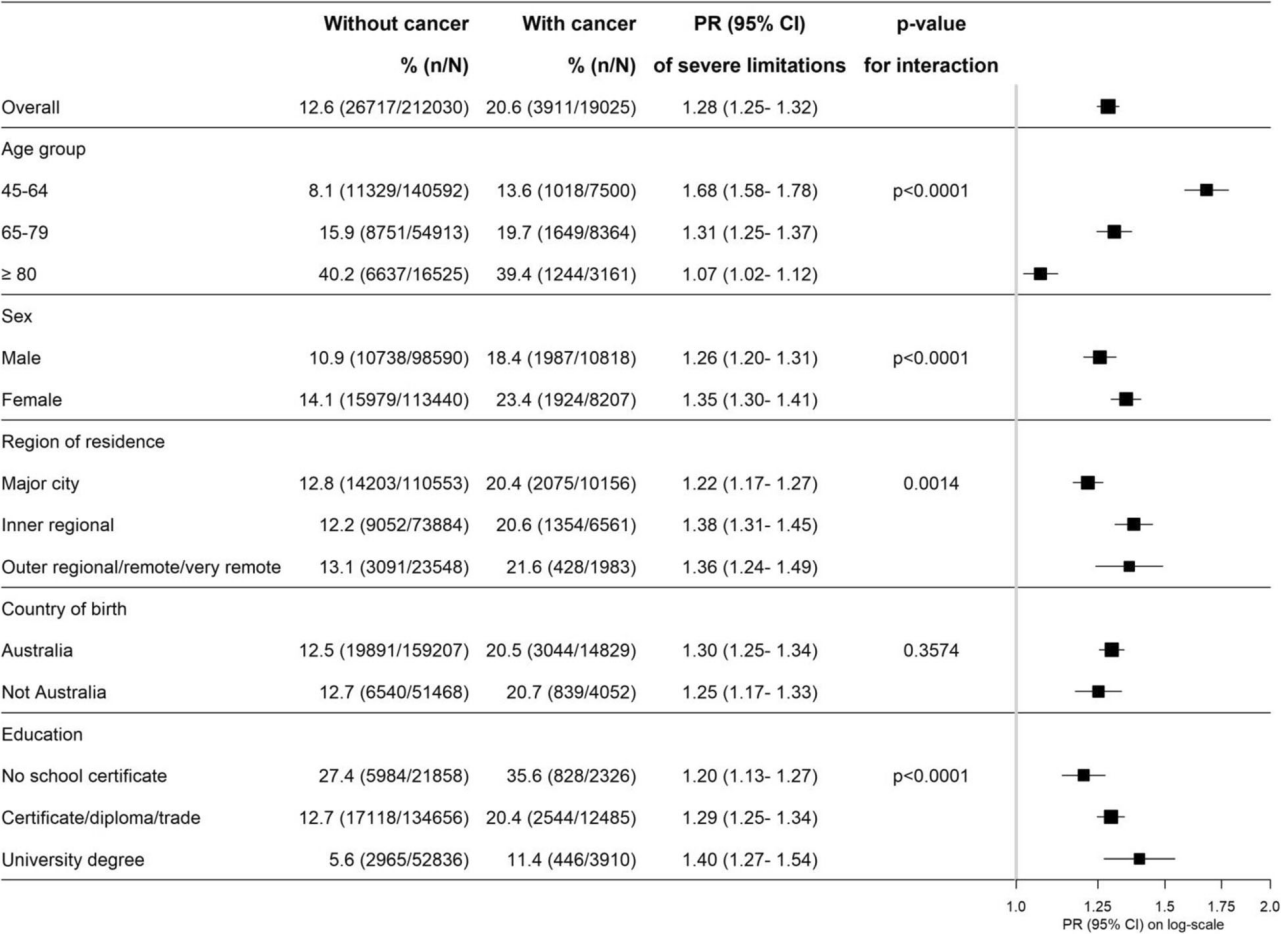
Prevalence of and adjusted prevalence ratios for severe physical functional limitations in a range of population subgroups

Worse physical functioning in those with compared to without cancer was observed in all of the demographic groups examined (Fig. 6) with the relation of cancer to severe physical functioning limitations much stronger in younger compared to older participants (Fig. 6, *p*_interaction_ < 0.0001), in parallel with increasing levels of physical disability with age among those without cancer. The relationship of cancer to severe physical functioning limitations was stronger for women compared to men (*P*_interaction_ < 0.0001), for those outside of major cities compared to those living in major cities (*p*_interaction_ = 0.0014) and for those with university education compared to those without school certificate (*p*_interaction_ < 0.0001). No significant difference was observed according to whether or not participants were born in Australia or elsewhere.

The overall pattern of elevated prevalences of adverse person-centred outcomes in cancer survivors compared to cancer-free individuals was similar when outcomes of moderate/severe limitations (MOS-PF < 90) and high distress (22 ≤ K-10 ≤ 50) were used (Additional file 2: Figs. S10-S11). When restricted to those without severe physical functioning limitations (Additional file 2: Fig. S12), the broad patterns remained similar, with attenuated effect estimates. Further adjustment for educational attainment did not materially change patterns of adverse outcomes across joint categories cancer and physical disability limitations (Additional file 2: Table S2).

## Discussion

In this large population-based study, compared to people without cancer, cancer survivors were more likely to report lower levels of physical functioning, self-rated health and quality of life and slightly higher psychological distress than those without cancer, with considerable variation across cancer types, time since diagnosis, treatments and stages.

Certain cancers such as lung cancer, multiple myeloma and the composite group of less common “other cancers” had the worst outcomes consistently across all the four measures investigated, while those with melanoma consistently reported similar levels to those without cancer across all outcome measures. The potential reasons for poor outcomes for some cancers include lower cure rate for some such as lung cancer (and thus ongoing symptoms related to cancer and its treatment), higher toxicity of treatment (such as bone marrow transplant commonly used in myeloma) and higher prevalence of comorbid disease (as in lung cancer and myeloma). The “other” cancer group is a heterogeneous group of rarer cancers, often with a lack of proven treatment protocols and with a range of prognoses and treatments, including some which are intense and toxic.

There are a number of factors that contribute to the variability of outcomes. Although the 5-year relative survival for people diagnosed with cancer has been improving over the last 30 years in Australia, survival is less than 50% for some cancer types such as multiple myeloma, mesothelioma, lung cancer, oesophagus cancer and pancreatic cancer. People diagnosed with these cancers are more likely to be living with incurable/metastatic cancer which adds to disability and emotional distress. Smoking, a key risk factor for many of these cancers, is also associated with other chronic conditions such as cardiovascular disease; living with multimorbidity adds to disability burden. This is also true for cancers with a higher incidence in older age groups (e.g. prostate cancer); despite improvements in screening and early detection, the burden of comorbid conditions at diagnosis is likely to contribute to poorer health and functioning. The favourable outcomes for melanoma may be explained by an earlier stage at diagnosis and relatively brief localised surgical treatment for the majority of cases.

Adverse person-centred outcomes attenuated with increasing time since diagnosis and for those with localised/unknown cancer stage and no recent treatment compared to others; psychological distress was not elevated among survivors five or more years post-diagnosis, with localised disease or without recent treatment. Overall, compared to those without cancer, cancer survivors had a significantly higher prevalence of physical functioning limitations in all of the population subgroups examined. Psychological distress and quality of life were much more strongly related to physical disability than to cancer diagnosis itself, with similar outcomes in cancer survivors and those without cancer, among people without physical disability. These are the first large-scale analyses, to our knowledge, to consider the relationship of cancer to psychological distress among people with and without physical disability, apart from the findings from an earlier subset of data from the 45 and Up Study [22]. The findings are broadly consistent with previous general studies on the importance of disability and physical morbidity in mental health [13] and evidence of increased distress with reduced physical functioning among women with breast cancer [23].

This is the most comprehensive study of person-centred outcomes across different cancer types, to our knowledge, considering multiple person-centred outcomes in the full range of common cancers and many less common cancer types, permitting comparisons across types and outcomes, including according to clinical factors and in different population subgroups. Previous studies have varied in terms of outcome measures used, study design, characteristics of cancer survivors and selection of controls; most have focused on single common cancer types such as breast and prostate cancer. Of the 104 studies identified, 29 studies [6–12, 22, 24–46] examined person-centred outcomes among survivors of different cancer types, and 75 studies [5, 23, 47–120] analysed survivors of a single cancer type (Additional file 3). In general, these studies have shown reduced physical functioning in cancer survivors compared to people without cancer and, where such data have been available, have also found variation according to cancer type and clinical characteristics. The likelihood of poor health and disability has also been shown to be higher among cancer survivors reporting comorbid chronic conditions [10]. Studies on mental health outcomes in those with versus without cancer have found comparable [7] to moderately elevated [22] adverse mental health outcomes in those with versus without cancer overall and a significantly higher likelihood of adverse outcomes for some rarer cancers [80]. Quality of life and self-rated health vary depending on the cancer type, but outcomes for long-term survivors generally approximate those of people without cancer. Limited evidence shows that person-centred outcomes vary according to time since diagnosis [121], therapeutic regimen [122], comorbidity [29] and cultural background [123].

This study was conducted using data from a large population-based study, with cancer diagnoses sourced from linked cancer registry data. The study questionnaire used validated measures of physical functioning limitations, psychological distress and self-rated health and a basic measure of quality of life. As in other cohort studies, the overall response rate to the baseline survey was modest (18%), and absolute prevalences in this cohort study may not be representative of the Australian population. However, cohort studies do not need to be representative to produce effect estimates—such as prevalence ratios—that are generalisable [124], and internal comparisons within cohort studies are generally reliable [125]. The study compares adverse person-centred outcomes in community-dwelling individuals with and without cancer. Although the possibility that some adverse outcomes could have been present before the onset of cancer cannot be excluded, it is not directly relevant to this comparison.

## Conclusions

The findings demonstrate the impact of and great variation in person-centred outcomes throughout the cancer journey and according to cancer type. They show the centrality of physical disability in relation to a person’s mental health and quality of life, both with and without cancer, and the need to support physical functioning, including by focusing on non-cancer morbidity [126]. These, in turn, emphasise the importance of holistic, integrated health—including by non-cancer providers such as general practitioners—in delivering the diversity of care required to optimise survivorship outcomes.

This study shows that physical disability is likely to be a key driver of psychological distress and reduced quality of life. In addition to routine screening for psychological distress [127], management of physical functioning and other symptoms is important in cancer survivorship. The positive long-term outcomes for breast cancer, even in the presence of physical disability, provide foundations for support and interventions for other cancer types. The study also shows the value of data on person-centred outcomes for quantifying cancer outcomes, in addition to standard measures such as mortality and health services use, including providing evidence to support planning and improvements in the provision of care. Ideally, assessment of person-centred outcomes including physical impairment should be part of the routine clinical assessment of cancer survivors at key time points such as completion of treatment and routine follow-up visits. The Model of Cancer Survivorship Care [128] recommended by the Clinical Oncology Society of Australia recognises this, but such assessment has not yet been adopted into routine clinical practice.

The evidence from this paper is also likely to be of use to cancer survivors and those supporting them, particularly in informing broad expectations for the cancer journey. The data highlight greater vulnerability and support needs around the time of diagnosis and treatment, with advanced disease and specific cancer types. They also provide reassurance that, for the majority of cancer types, mental health and quality of life in longer-term survivors do not differ markedly from that in people without cancer. This mirrors qualitative studies which demonstrate that after an adjustment period, many cancer survivors report that they are coping well, are managing and adapting to any ongoing symptoms/side effects and have found a “new normal” [129, 130]. Although access to specific survivorship care services within the Australian health care system is limited and variable, as is the case in most countries around the world [131], cancer survivors are able to draw on universal primary, secondary and tertiary care in Australia. Such care is likely to contribute in general terms to outcomes in survivors, although shortfalls in these services have been noted.

There is a need to consider the diversity of cancers, including less common ones and those with poor survival, when investigating contributors to poor physical function and identifying targets for intervention; these contributors and targets are likely to include cancer progression, risk factors and treatment, as well as comorbid conditions, contextual factors and life circumstances.

## Supplementary Information

**Additional file 1.** : Brief Summary of Consumer Engagement Plan.

**Additional file 2: Table S1.** Number of cases grouped under “other” cancers. **Table S2.** Prevalence of and age-, sex- and education- adjusted prevalence ratios (PRs) for adverse person-centred outcomes according to joint categories of physical functioning limitations and cancer. **Figure S1.** Prevalence of moderate/high distress by cancer type and time since diagnosis. **Figure S2.** Prevalence of moderate/high distress by cancer type and stage. **Figure S3.** Prevalence of moderate/high distress by cancer type and recent treatment. **Figure S4.** Prevalence of poor/fair self-rated health by cancer type and time since diagnosis; **Figure S5.** Prevalence of poor/fair self-rated health by cancer type and stage. **Figure S6.** Prevalence of poor/fair self-rated health by cancer type and recent treatment; **Figure S7.** Prevalence of poor/fair self-rated quality of life by cancer type and time since diagnosis. **Figure S8.** Prevalence of poor/fair self-rated quality of life by cancer type and stage. **Figure S9.** Prevalence of poor/fair self-rated quality of life by cancer type and recent treatment. **Figure S10.** Prevalence of high psychological distress by cancer type. **Figure S11.** Prevalence of moderate/severe physical functioning limitations by cancer type. **Figure S12.** Prevalence of and age- and sex- adjusted prevalence ratios (PR) for adverse person-centred outcomes by cancer type: analyses restricted to those without severe physical functioning limitations.

**Additional file 3.** : Brief review of person-centred outcomes and cancer.

## Data Availability

Data supporting the findings from this study are available from the Sax Institute, the NSW Department of Health and the Australian Bureau of Statistics, with data linkage conducted by the NSW Centre for Health Record Linkage. Restrictions apply to the availability of these data, which were used under licence for the current study, and so are not publicly available. Data are however available from the authors upon reasonable request and with permission from the Sax Institute (www.saxinstitute.org.au) and the NSW Department of Health.
