# Supplementary material for: Disability, psychological distress and quality of life in relation to cancer diagnosis and cancer type: population-based Australian study of 22,505 cancer survivors and 244,000 people without cancer
Source: BMC Med. 2020 Dec 1;18:372. doi: 10.1186/s12916-020-01830-4 (PMC7708114; doi:10.1186/s12916-020-01830-4)
Supplement: Supplementary file 1 — Additional file 1. : Brief Summary of Consumer Engagement Plan. [file 12916_2020_1830_MOESM1_ESM.docx]

# ADDITIONAL FILE 1: Brief Summary of Consumer Engagement Plan

This paper is part of a larger project which was developed and is being conducted in partnership with consumers. The project is entitled *Large-scale data to understand person-centred outcomes in cancer survivors* (funded by the National Health and Medical Research Council of Australia). Initial scoping consultation was undertaken with a Cancer Research Consumer Representatives Group from the Australian National University in 2015, informed by quantitative and qualitative research on consumer priorities. The project and funding application were developed with John Stubbs, the CEO of Cancer Voices Australia as an investigator, working closely with the other investigators. Following funding, the project commenced on 1 Jan 2018.

A formal consumer engagement plan was drafted for the project after funding, including the outlining of the roles of researchers and consumers at each phase of the project. A literature review informed the plan, resulting in the choice of an overall model drawing on that of the South Australian Health and Medical Research Institute’s Consumer and Community Engagement Framework. The research project has three broad phases: preparation, research conduct and implementation. Consumers were involved during the preparation phase, which is now complete, as the project has been awarded funding and is underway. This research project is currently in the execution phase, so the bulk of the plan is related to research conduct and implementation.

Consumer groups involved in this project include:

1. John Curtin School of Medical Research’s Cancer Research Consumer Representatives Group
2. Joint Community Advisory Group, a joint initiative of the Psycho-oncology Cooperative Research Group of the University of Sydney and the Primary Care Collaborative Cancer Clinical Trials Group
3. Psycho-oncology Cooperative Research Group Community Advisory Group of the University of Sydney

Principal and other consumers engaged with the project attend investigator meetings, provide open and frank feedback and guidance on all aspects of the research, provide input to any potential papers that meet their interests and provide leadership in dissemination and/or implementation, where it suits their interests and capacity. For this large and complex project, the phases will run concurrently, integrating the conduct of research and its implementation, where relevant, into policy and practice.
