# Supplementary material for: Disability, psychological distress and quality of life in relation to cancer diagnosis and cancer type: population-based Australian study of 22,505 cancer survivors and 244,000 people without cancer
Source: BMC Med. 2020 Dec 1;18:372. doi: 10.1186/s12916-020-01830-4 (PMC7708114; doi:10.1186/s12916-020-01830-4)

**ADDITIONAL FILE 2: SUPPLEMENTARY TABLES AND FIGURES**

[Table S1. Number of cases grouped under “other” cancers. 2](#_Toc52197611)

[Table S2. Prevalence of and age-, sex- and education- adjusted prevalence ratios (PRs) for adverse person-centred outcomes according to joint categories of physical functioning limitations and cancer 3](#_Toc52197612)

[Figure S1. Prevalence of moderate/high distress by cancer type and time since diagnosis 4](#_Toc52197613)

[Figure S2. Prevalence of moderate/high distress by cancer type and stage 5](#_Toc52197614)

[Figure S3. Prevalence of moderate/high distress by cancer type and recent treatment 6](#_Toc52197615)

[Figure S4. Prevalence of poor/fair self-rated health by cancer type and time since diagnosis 7](#_Toc52197616)

[Figure S5. Prevalence of poor/fair self-rated health by cancer type and stage 8](#_Toc52197617)

[Figure S6. Prevalence of poor/fair self-rated health by cancer type and recent treatment 9](#_Toc52197618)

[Figure S7. Prevalence of poor/fair self-rated quality of life by cancer type and time since diagnosis 10](#_Toc52197619)

[Figure S8. Prevalence of poor/fair self-rated quality of life by cancer type and stage 11](#_Toc52197620)

[Figure S9. Prevalence of poor/fair self-rated quality of life by cancer type and recent treatment 12](#_Toc52197621)

[Figure S10. Prevalence of high psychological distress by cancer type 13](#_Toc52197622)

[Figure S11. Prevalence of moderate/severe physical functioning limitations by cancer type 14](#_Toc52197623)

[Figure S12. Prevalence of and age- and sex- adjusted prevalence ratios (PR) for adverse person-centred outcomes by cancer type: analyses restricted to those without severe physical functioning limitations 15](#_Toc52197624)

## Table S1. Number of cases grouped under “other” cancers.

| **ICD-10AM Code** | **ICD-10AM label** | **n** |
| --- | --- | --- |
| C00 | Lip | 178 |
| C01-C02 | Tongue | 92 |
| C03-C06 | Mouth | 76 |
| C07-C08 | Salivary glands | 54 |
| C09-C10 | Oropharynx | 65 |
| C11 | Nasopharynx | 23 |
| C12-C13 | Hypopharynx | 12 |
| C14 | Other sites in pharynx | 9 |
| C16 | Stomach | 167 |
| C17 | Small intestine | 70 |
| C21 | Anus | 56 |
| C22 | Liver | 43 |
| C23-C24 | Gallbladder and extrahepatic bile ducts | 47 |
| C25 | Pancreas | 52 |
| C26 | Other digestive organs | 2 |
| C30-C31 | Nose, sinuses, etc | 18 |
| C32 | Larynx | 119 |
| C37-C39 | Other thoracic and respiratory organs | 7 |
| C40-C41 | Bone | 28 |
| C42.1 | Bone Marrow | 1 |
| C45 | Mesothelioma | 25 |
| C46 | Kaposi sarcoma | 11 |
| C48 | Peritoneum | 28 |
| C47, C49 | Other soft tissue | 84 |
| C51 | Vulva | 37 |
| C52 | Vagina | 6 |
| C53 | Cervix | 88 |
| C56 | Ovary | 189 |
| C57-C58 | Other female genital organs and placenta | 14 |
| C60 | Penis | 13 |
| C62 | Testis | 77 |
| C63 | Other male genital organs | 7 |
| C65-C66, C68 | Other urinary organs | 55 |
| C69 | Eye | 64 |
| C71 | Brain | 47 |
| C70, C72 | Other central nervous system | 9 |
| C74-C75 | Other endocrine glands | 8 |
| C76 | Other and ill-defined sites | 4 |
| C80 | Unknown primary site | 121 |
| C81 | Hodgkin's disease | 64 |
| C88 | Immunoproliferative cancers | 8 |
| C90.1-C90.9 | Other plasma cell | 15 |
| C96, D45, D47.1, D47.3-D47.5 | Other cancers of the blood and lymphatic system | 95 |
| D46 | Myelodysplastic syndromes | 74 |
| Total |  | 2262 |

## Table S2. Prevalence of and age-, sex- and education- adjusted prevalence ratios (PRs) for adverse person-centred outcomes according to joint categories of physical functioning limitations and cancer

|  | **moderate/high distress** | | **poor/fair self-rated health** | | **poor/fair self-rated quality of life** | |
| --- | --- | --- | --- | --- | --- | --- |
|  | **%(n/N)** | **PR (95% CI)** | **%(n/N)** | **PR (95% CI)** | **%(n/N)** | **PR (95% CI)** |
| Without cancer | 23.1(45357/196185) | 1 | 12.5(26122/209129) | 1 | 9.4(19478/206394) | 1 |
| With cancer | 22.2(3746/16915) | 1.06(1.02-1.09) | 20.7(3851/18651) | 1.45(1.41-1.50) | 14.0(2579/18444) | 1.30(1.25-1.35) |
| No cancer and |  |  |  |  |  |  |
| No limitations | 15.7(11081/70776) | 1 | 2.1(1552/73819) | 1 | 2.6(1897/72689) | 1 |
| Minor limitations | 18.3(10591/57822) | 1.28(1.25-1.31) | 4.5(2754/60606) | 2.24(2.10-2.38) | 3.5(2093/59896) | 1.37(1.29-1.46) |
| Moderate limitations | 28.5(12860/45149) | 2.12(2.08-2.17) | 16.7(8146/48743) | 8.48(8.03-8.95) | 10.5(5085/48234) | 4.19(3.98-4.42) |
| Severe limitations | 48.2(10825/22438) | 3.73(3.65-3.81) | 52.7(13670/25961) | 26.47(25.12-27.90) | 40.7(10403/25575) | 15.77(15.02-16.56) |
| Cancer and |  |  |  |  |  |  |
| No limitations | 12.1(457/3768) | 0.87(0.80-0.95) | 2.7(108/4012) | 1.28(1.05-1.56) | 2.6(101/3946) | 0.97(0.80-1.19) |
| Minor limitations | 13.3(634/4765) | 1.11(1.03-1.20) | 5.4(276/5089) | 2.75(2.42-3.12) | 3.6(179/5036) | 1.43(1.23-1.67) |
| Moderate limitations | 23.6(1231/5213) | 2.08(1.97-2.19) | 21.1(1218/5768) | 10.70(9.91-11.55) | 12.0(686/5714) | 4.84(4.42-5.30) |
| Severe limitations | 44.9(1424/3169) | 3.99(3.81-4.17) | 59.5(2249/3782) | 29.30(27.43-31.30) | 43.0(1613/3748) | 16.70(15.56-17.92) |

## Figure S1. Prevalence of moderate/high distress by cancer type and time since diagnosis


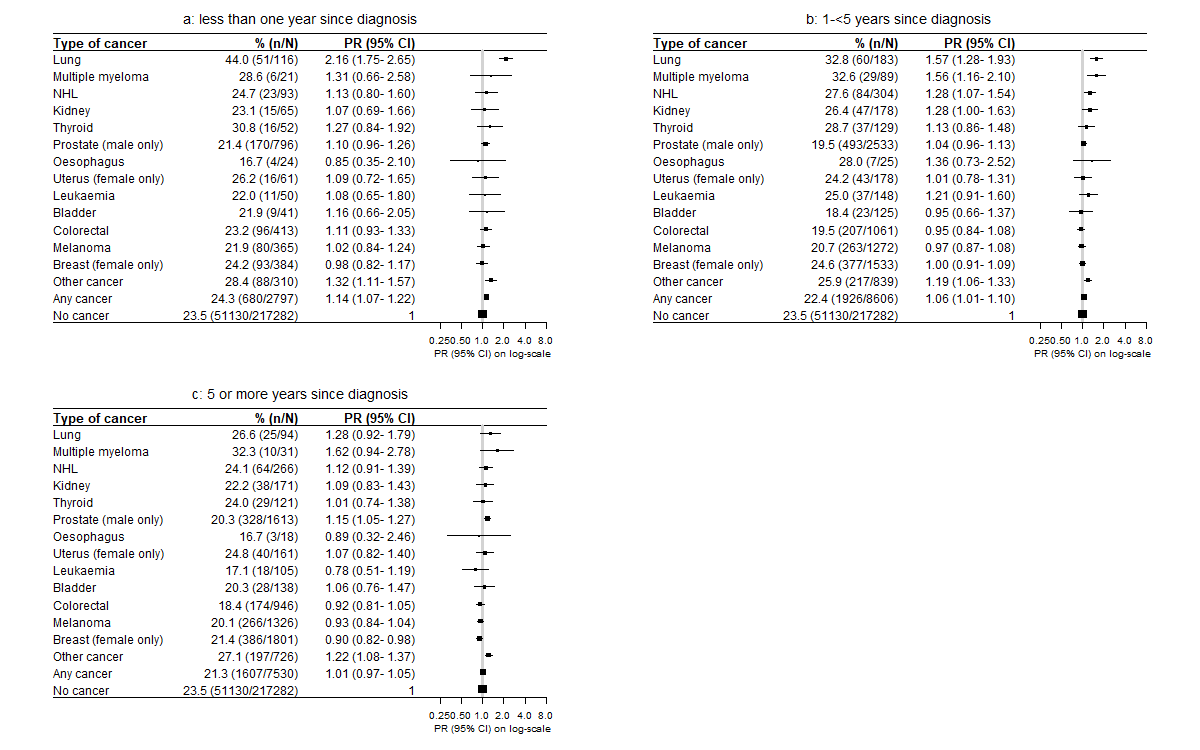


Figure S2. Prevalence of moderate/high distress by cancer type and stage
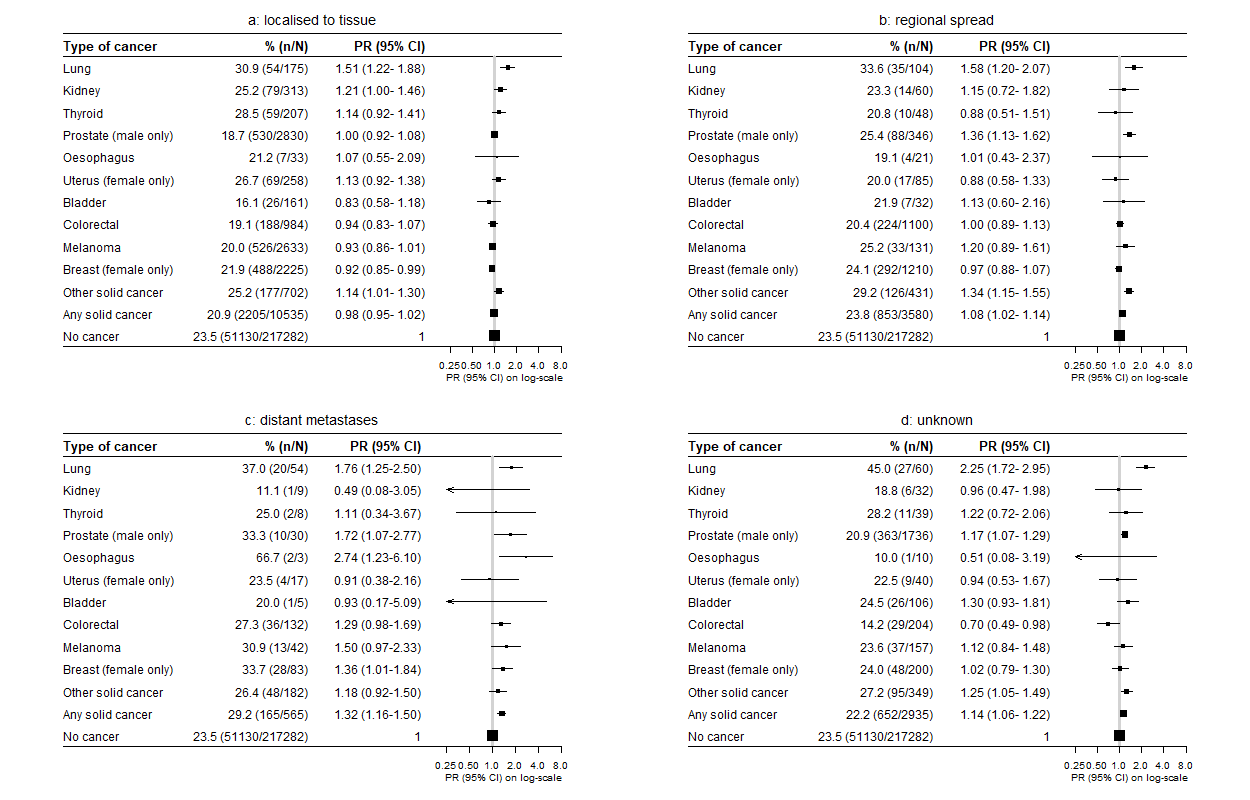


## Figure S3. Prevalence of moderate/high distress by cancer type and recent treatment


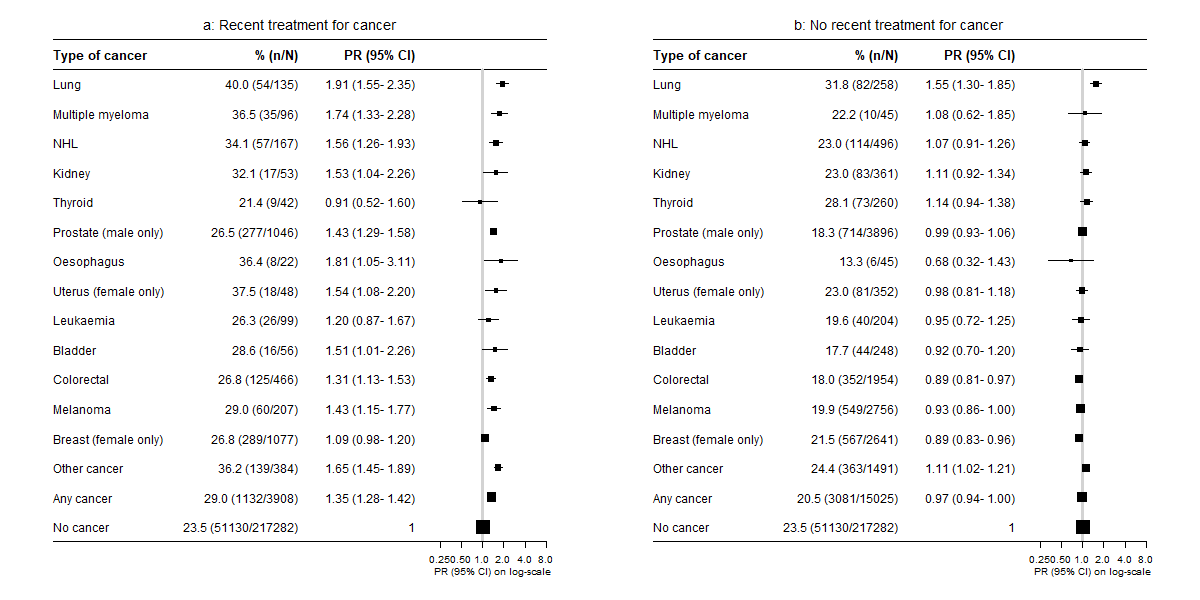


Figure S4. Prevalence of poor/fair self-rated health by cancer type and time since diagnosis
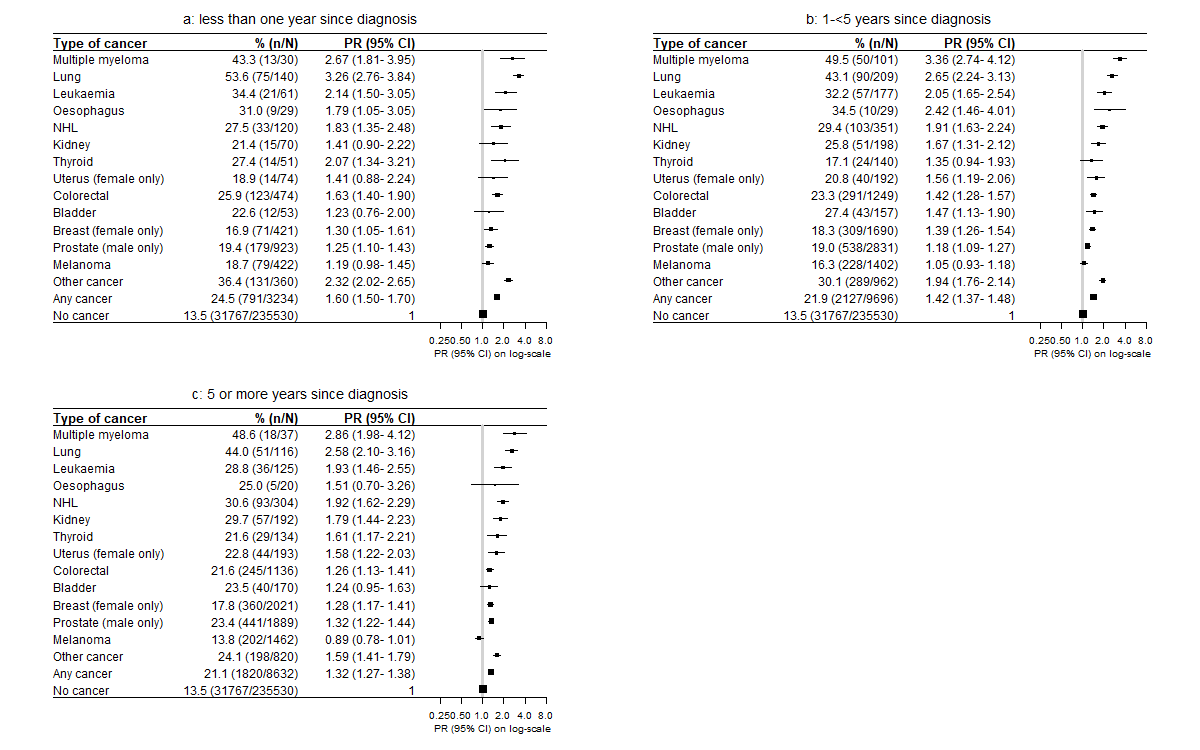


## Figure S5. Prevalence of poor/fair self-rated health by cancer type and stage


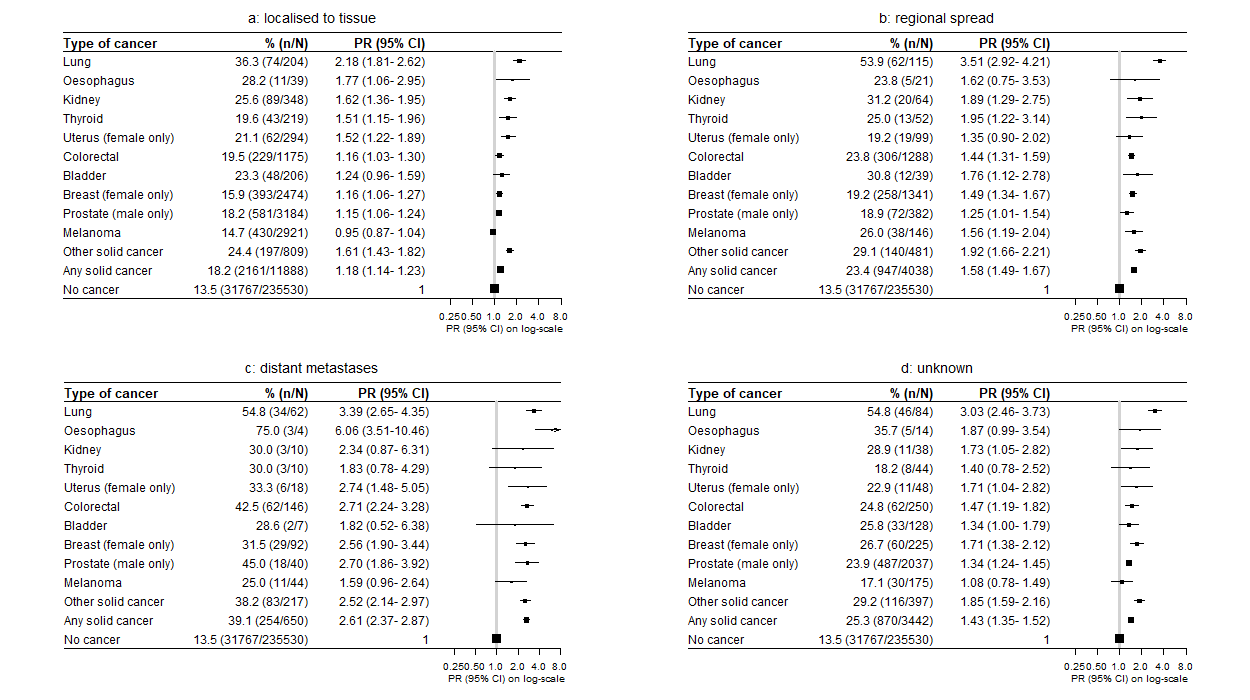


## Figure S6. Prevalence of poor/fair self-rated health by cancer type and recent treatment


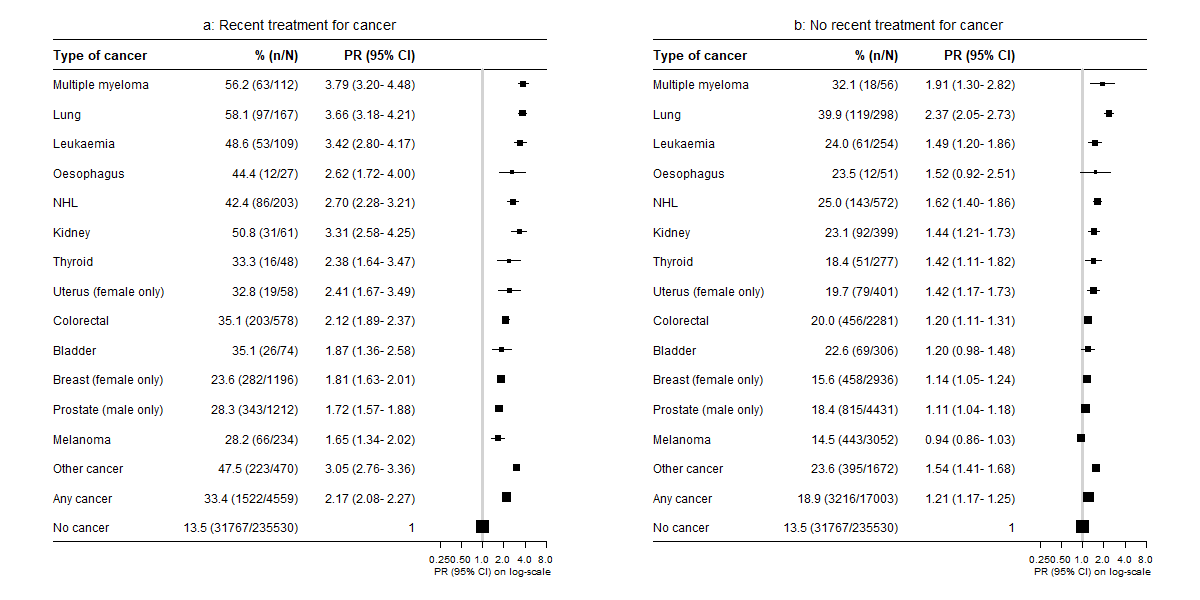


## Figure S7. Prevalence of poor/fair self-rated quality of life by cancer type and time since diagnosis


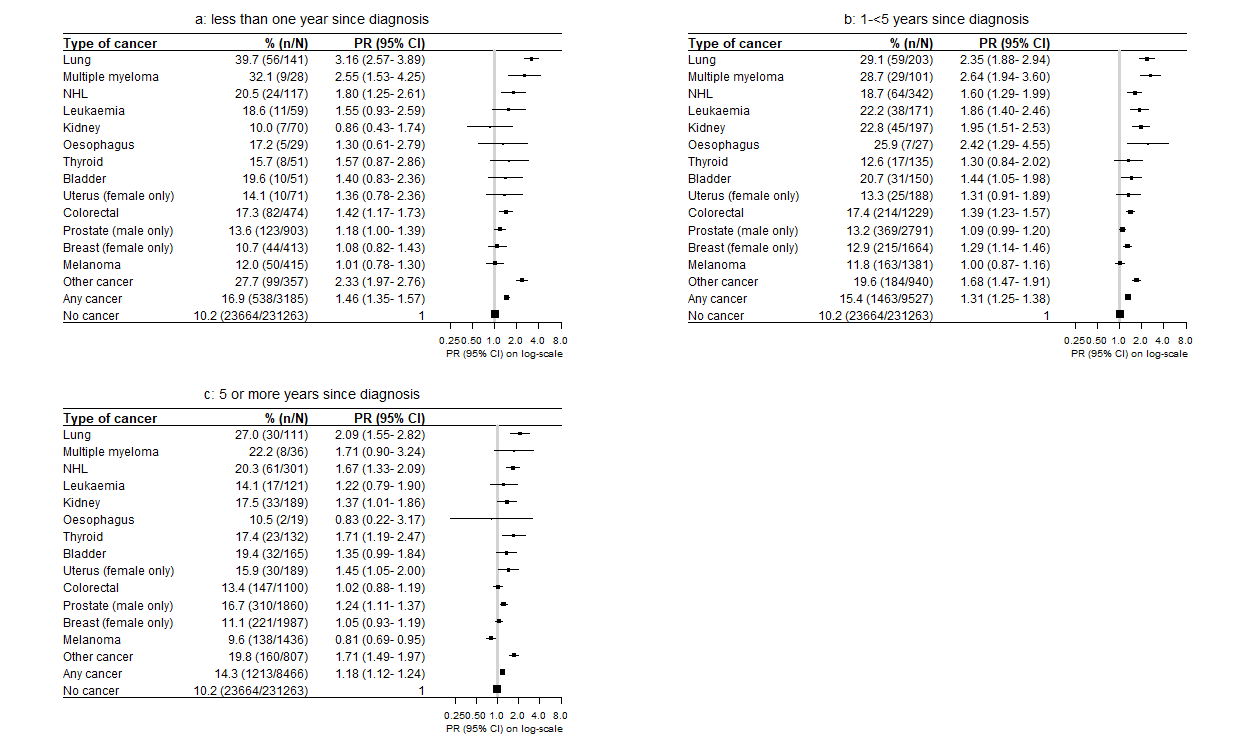


Figure S8. Prevalence of poor/fair self-rated quality of life by cancer type and stage
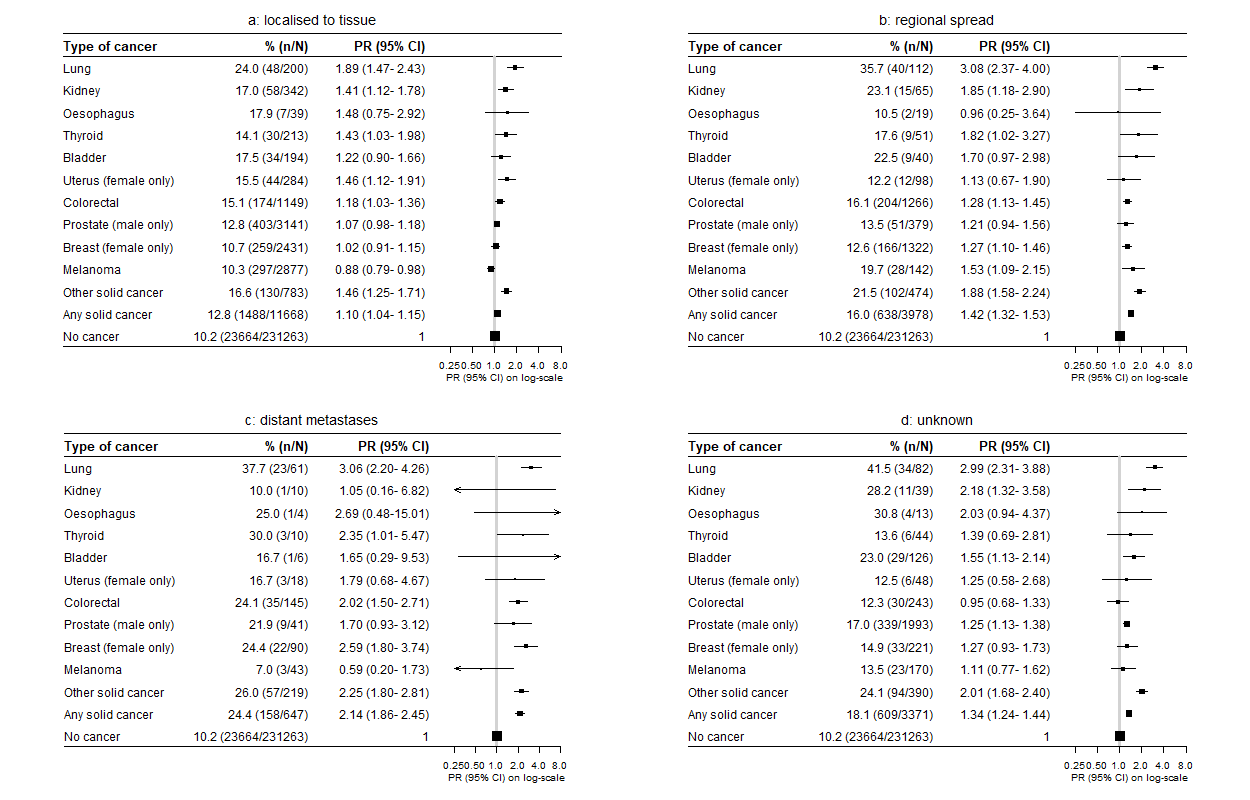


## Figure S9. Prevalence of poor/fair self-rated quality of life by cancer type and recent treatment


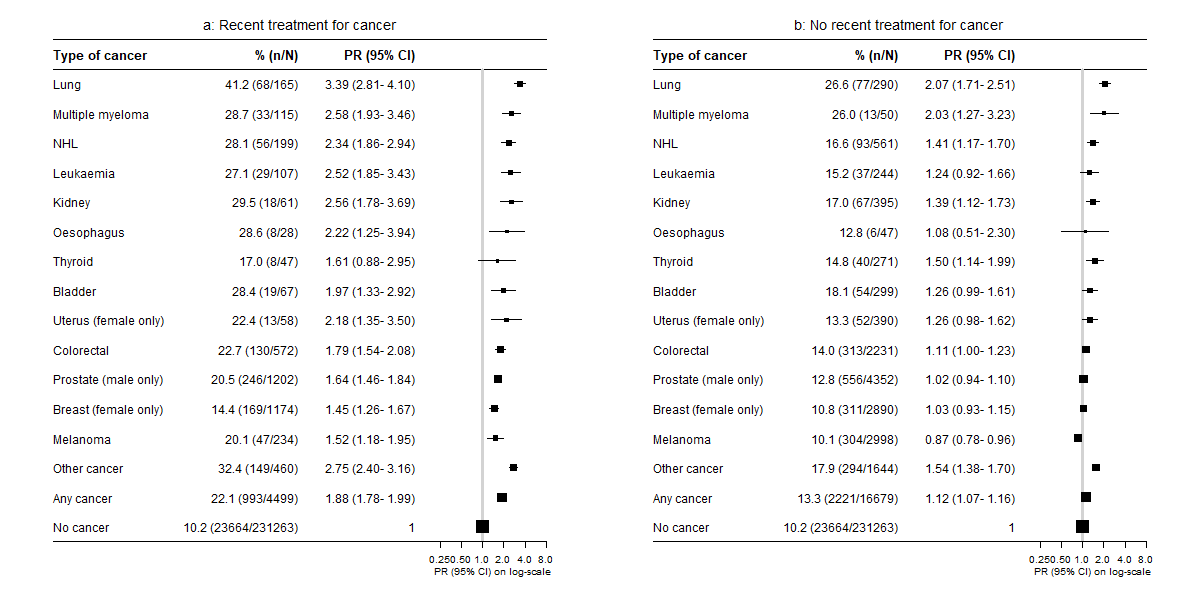


## Figure S10. Prevalence of high psychological distress by cancer type


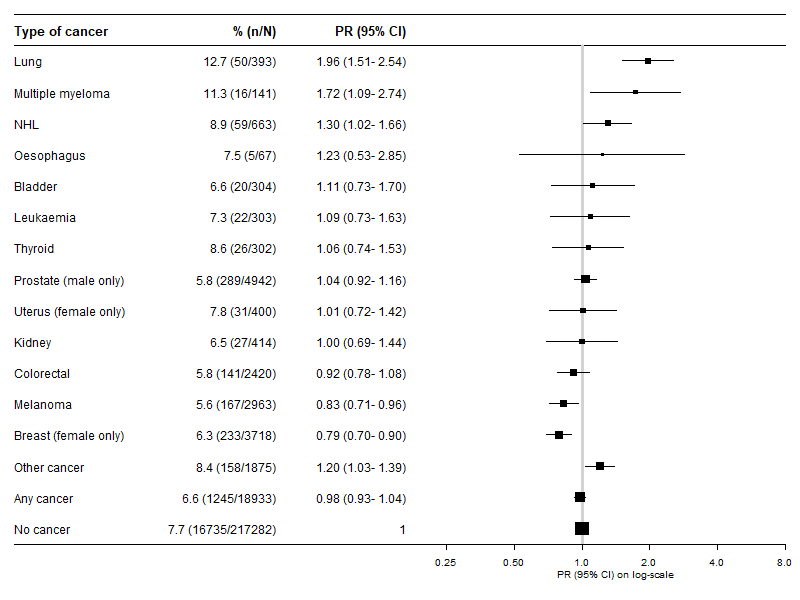


## Figure S11. Prevalence of moderate/severe physical functioning limitations by cancer type


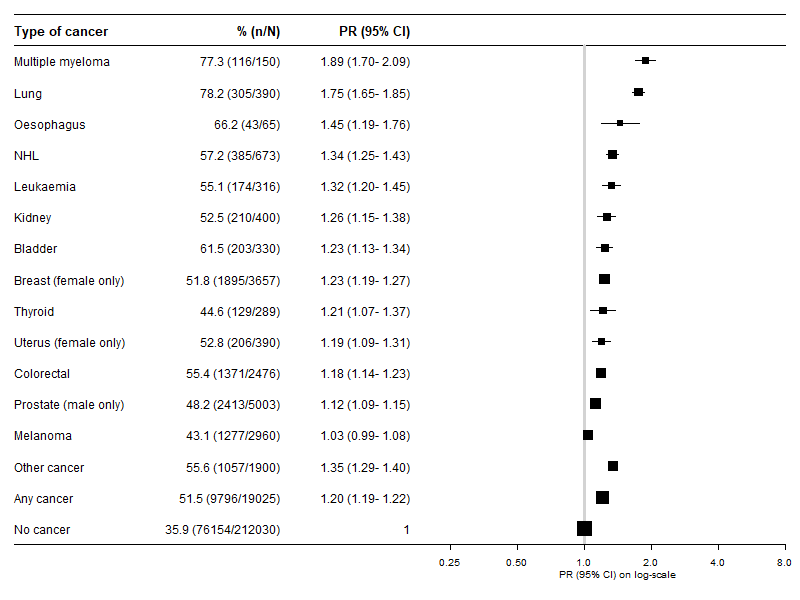


## Figure S12. Prevalence of and age- and sex- adjusted prevalence ratios (PR) for adverse person-centred outcomes by cancer type: analyses restricted to those without severe physical functioning limitations


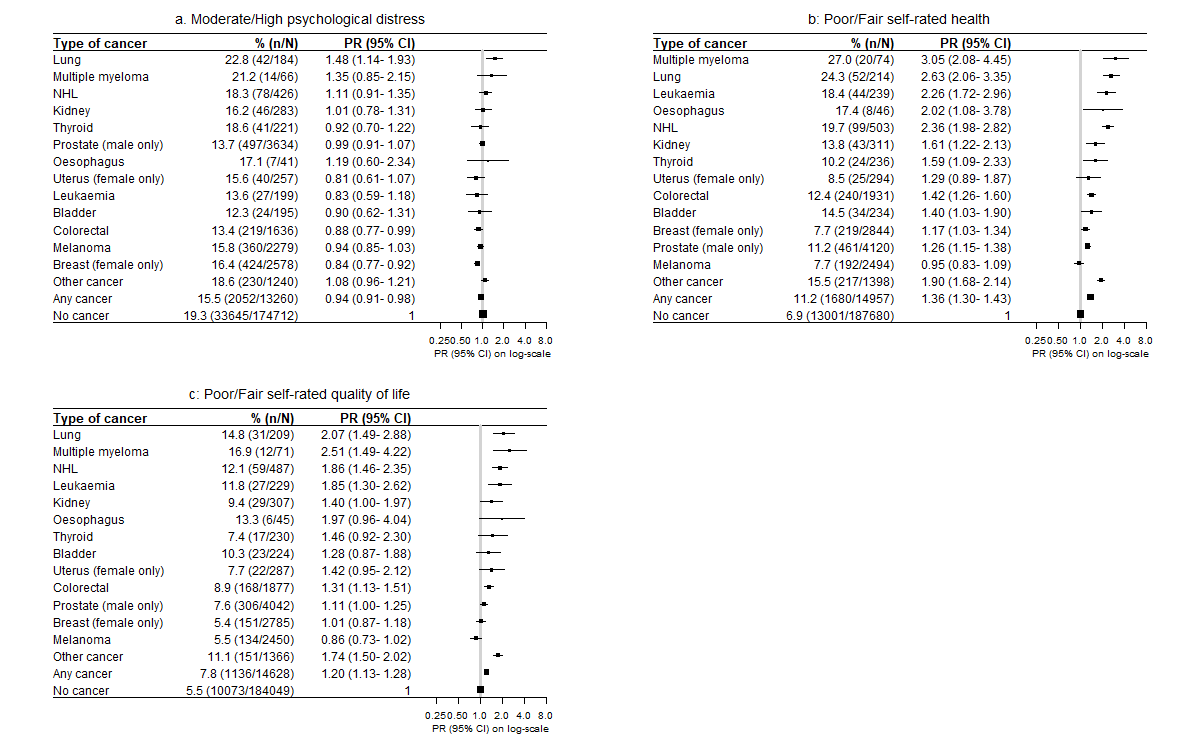

Supplement: Supplementary file 2 — Additional file 2: Table S1. Number of cases grouped under “other” cancers. Table S2. Prevalence of and age-, sex- and education- adjusted prevalence ratios (PRs) for adverse person-centred outcomes according to joint categories of physical functioning limitations and cancer. Figure S1. Prevalence of moderate/high distress by cancer type and time since diagnosis. Figure S2. Prevalence of moderate/high distress by cancer type and stage. Figure S3. Prevalence of moderate/high distress by cancer type and recent treatment. Figure S4. Prevalence of poor/fair self-rated health by cancer type and time since diagnosis; Figure S5. Prevalence of poor/fair self-rated health by cancer type and stage. Figure S6. Prevalence of poor/fair self-rated health by cancer type and recent treatment; Figure S7. Prevalence of poor/fair self-rated quality of life by cancer type and time since diagnosis. Figure S8. Prevalence of poor/fair self-rated quality of life by cancer type and stage. Figure S9. Prevalence of poor/fair self-rated quality of life by cancer type and recent treatment. Figure S10. Prevalence of high psychological distress by cancer type. Figure S11. Prevalence of moderate/severe physical functioning limitations by cancer type. Figure S12. Prevalence of and age- and sex- adjusted prevalence ratios (PR) for adverse person-centred outcomes by cancer type: analyses restricted to those without severe physical functioning limitations. [file 12916_2020_1830_MOESM2_ESM.docx]
