# Supplementary material for: Disability, psychological distress and quality of life in relation to cancer diagnosis and cancer type: population-based Australian study of 22,505 cancer survivors and 244,000 people without cancer
Source: BMC Med. 2020 Dec 1;18:372. doi: 10.1186/s12916-020-01830-4 (PMC7708114; doi:10.1186/s12916-020-01830-4)
Supplement: Supplementary file 3 — Additional file 3. : Brief review of person-centred outcomes and cancer. [file 12916_2020_1830_MOESM3_ESM.docx]

# Additional File 3: Brief review of person-centred outcomes and cancer

**Aim:** To summarise the worldwide evidence relevant to our findings on the physical and mental health-related outcomes in people living with versus without cancer.

**Description of Methods:** We focused initially on large-scale studies considering multiple cancers and assessing outcomes similar to those investigated in this study. We included published individual studies providing relative estimates of outcomes (relative to those without cancer); this included those with multiple, unspecified and single cancers types as well as a number of studies without a cancer-free comparison group. For studies based on common cancer types (breast or colorectal cancer survivors), samples sizes greater than 500 were given priority during synthesis. Manual searching of reference lists was undertaken to locate additional publications. Reference lists of included studies were searched, as well as studies included in relevant recent systematic reviews and meta-analyses.

**Interpretation:** The list of identified studies is given in the reference section below. The results of the studies identified in the review are compared with those of our study, in the discussion section of the main paper. Studies comparing the prevalence of adverse person-centred outcomes (specifically self-rated health, self-reported quality of life, psychological distress, and physical function limitations) in cancer survivors with those who do not have cancer are emphasised, as these are the comparators in our study.

**Outcomes:** Person-centred outcomes, specifically self-rated health, self-reported quality of life, psychological distress and physical function limitations

**Inclusion criteria:**

Study designs: Published, peer-reviewed studies

Population: Individuals with cancer diagnosis

Comparison: Individuals without cancer diagnosis (general population)

Outcome: Person-centred outcomes including psychological distress, physical function limitations, self-rated health and self-reported quality of life.

Timing: All years

Setting: Any country

Language: Articles reported in English.

**Exclusion criteria:**

Study designs: Grey literature, conference abstracts, letters, editorials, correspondence, opinion pieces, government reports, position statements

Population: Individuals without a cancer diagnosis and childhood cancer survivors

Outcome: Outcomes other than person-centred outcomes such as clinical outcomes, and studies that present data on variables associated with person centred outcomes

Timing: No exclusion criteria

Setting: No exclusion criteria

Language: Articles not published in English

**Search strategy:**

Population terms

1. Cancer survivor
2. Cancer survivors
3. Cancer patient
4. Cancer patients
5. Survivor
6. Survivors
7. Long-term survivor
8. Long-term survivors

Cancer specific terms (combined with above terms)

1. Breast
2. Colorectal
3. Kidney
4. Leukaemia
5. Lung
6. Melanoma
7. Multiple myeloma
8. NHL
9. Oesophagus
10. Prostate
11. Thyroid
12. Uterus

Outcome terms included in search:

1. Health related quality of life
2. Quality of life
3. Self-rated health
4. Self-assessed health status
5. Self-reported wellbeing
6. Self-reported quality of life
7. Self-reported general health
8. Physical functioning
9. Physical function limitation
10. Physical performance
11. Physical activity
12. Psychological distress
13. Mental health
14. Mental wellbeing
15. Depression
16. Anxiety
17. Person centred outcomes
18. Patient centred outcomes
19. Patient reported outcomes

**Databases:** Pubmed, Google Scholar and ANU Super Search were searched to identify relevant literature. The reference lists of relevant articles were searched and the cited by function employed to identify any other studies. Databases were search between April and June 2019.

**Screening:** Articles were title screened and selected for abstract review if they contained a phrase similar to ‘cancer survivor’ in combination with a person-centred outcome. During abstract screening, details pertaining to outcomes and comparators and were identified with the full text of relevant articles reviewed. If there was insufficient detail to make a judgement on the relevance of the article, the full text was reviewed. Articles that met all the inclusion criteria at full text screening were included in the literature review.

**Studies identified from the search:** The full texts of 104 papers were reviewed; these included two U.S studies using the National Health Interview Survey and one Australian study based on the same cohort contributing to manuscript. 29 studies (6-12, 22, 24-46) examined person-centred outcomes among survivors of different cancer types and 75 studies (5, 23, 47-120) analysed survivors of a single cancer type.
